# Supplementary material for: Determinants of hyperemesis gravidarum among pregnant women attending health care service in public hospitals of Southern Ethiopia
Source: PLoS One. 2022 Apr 26;17(4):e0266054. doi: 10.1371/journal.pone.0266054 (PMC9042275; doi:10.1371/journal.pone.0266054)
Supplement: S1 File — (DOCX) [file pone.0266054.s001.docx]

**English version of the questionaries’**

**Part one: Socio-demographic characteristics related questions**

| S/n | Variables | Response |
| --- | --- | --- |
| 1 | What is your age in year? | _______ |
| 2 | What is your residence? | 1. Urban 2. Rural |
| 3 | What is your ethnicity? | 1. Gamo 2. Gofa 3. Welayita 4. Konso 5. Omo 6. Others |
| 4 | What is your religion? | 1. Orthodox 2. Muslim 3. Protestant 4. Others |
| 5 | Your marital status? | 1. Single 2. Married 3. Separated 4. Divorced 5. Widowed |
| 6 | Do you have polygyny husband? | 1. Yes 2. No |
| 8 | Your occupation? | 1. Housewife 2. Student 3. Merchant 4. Government employee 5. Farmer 6. Daily laborer 7. Other |
| 9 | Educational status? | 1. No formal education 2. 1–8th 3. 9–12th 4. College and above |
| 10 | How often you do leisure time physical activity(running, walking, bicycling, aerobic dancing, jumping jacks, climbing stairs, and jumping rope)? | ______________ |

**Part two: Reproductive characteristics related questions**

| S/n | Variables | Response |
| --- | --- | --- |
| 1 | Gestational age in week? | ________ week |
| 2 | How many times do you get pregnant? | ________ |
| 3 | How many times have you given birth? | 1. Nulliparous 2. Primiparous 3. Multiparous |
| 4 | How many live children do you have? | _____ |
| 5 | Have you encountered previous multiple pregnancies? | 1. Yes 2. No |
| 6 | How far is the time between the previous and current pregnancy in a month? | __________ |
| 7 | Do you encounter molar pregnancy (snow storm/ grape like) confirmed by physician in a previous pregnancy or and if documented? | 1. Yes 2. No |
| 8 | Do you encounter gestational hypertension in a previous pregnancy and if documented? | 1. Yes 2. No |
| 9 | Do you encounter hyperemesis gravidarum in a previous pregnancy and if documented? | 1. Yes 2. No |
| 10 | Is your pregnancy planed (occurred in the desired time agreed by you and your partner)? | 1. Yes 2. No |
| 11 | Is your pregnancy wanted (were you and your partner had desire of children)? | 1. Yes 2. No |
| 12 | Is your pregnancy supported? (Did you get psychological/economical supports and care by the partner) for your pregnancy? | 1. Yes 2. No |
| 13 | If you were pregnant, did you encounter any one of bad obstetric outcome (abortion, preterm delivery, fetal anomaly, neonatal death, fetal growth restriction, neural tube defect) and if documented? | 1. Yes 2. No |

**Part three: maternal medical factors related**

| S/N | Variables | | | Response | | |
| --- | --- | --- | --- | --- | --- | --- |
| 1 | Have you been diagnosed with diabetes mellitus before you get pregnant and if documented? | | | 1. Yes 2. No | | |
| 2 | Have you been diagnosed with hyperthyroid disorder before pregnancy or if documented? | | | 1. Yes 2. No | | |
| 3 | Have you got asthma/other respiratory problems before pregnancy and if documented? | | | 1. Yes 2. No | | |
| 4 | Have you experienced pre-pregnancy motion-sickness? | | | 1. Yes 2. No | | |
| 5 | Depression | Not at all | Several days | | More than half the days | Nearly every day |
|  | How often you have little interest or pleasure in doing things? |  |  | |  |  |
|  | How often you feel down, depressed or hopeless? |  |  | |  |  |
|  | How often you encounter trouble with falling or staying asleep or sleeping too much? |  |  | |  |  |
|  | How often you feel tired or have little energy? |  |  | |  |  |
|  | How often you have a poor appetite or overeating? |  |  | |  |  |
|  | How often you feel bad about yourself or that you are a failure or have let yourself or your family down? |  |  | |  |  |
|  | How often you encounter trouble concentrating on things, such as reading the newspaper or watching television? |  |  | |  |  |
|  | How often you encounter moving or speaking so slowly that other people could have noticed?  Or the opposite – being so fidgety or restless that you have been moving around a lot more than usual? |  |  | |  |  |
|  | How often you thought that you would be better off dead or hurting yourself in some way? |  |  | |  |  |

**Part four: psychological factors**

| SN | Variables | Response | | | | |
| --- | --- | --- | --- | --- | --- | --- |
| 1 | **Perceived stress illness** | Never | Almost never | Sometimes | Fairly often | Very often |
|  | In the last month, how often have you been upset because of something that happened unexpectedly? |  |  |  |  |  |
|  | In the last month, how often have you felt that you were unable to control the important things in your life? |  |  |  |  |  |
|  | In the last month, how often have you felt nervous and stressed? |  |  |  |  |  |
|  | In the last month, how often have you felt confident about your ability to handle your personal problems? |  |  |  |  |  |
|  | In the last month, how often have you felt that things were going your way? |  |  |  |  |  |
|  | In the last month, how often have you found that you could not cope with all the things that you had to do? |  |  |  |  |  |
|  | In the last month, how often have you been able to control irritations in your life? |  |  |  |  |  |
|  | In the last month, how often have you felt that you were on top of things? |  |  |  |  |  |
|  | In the last month, how often have you been angered because of things that happened that been outside of your control? |  |  |  |  |  |
|  | In the last month, how often have you felt difficulties were piling up so high that you could not overcome them? |  |  |  |  |  |
| 2 | If you have history of bad obstetrics history do you afraid that the event you encountered will occur again in the current pregnancy? | 1. Yes 2. No | | | | |

**Part five: dietary related variables**

| SN | Variables | Response |
| --- | --- | --- |
| 1 | How often do you drink milk in the current pregnancy, till the day you visit this hospital? | 1. Daily 2. __________per week 3. __________ per 2 week 4. __________ per 3 week 5. ___________ per month 6. Not at all |
| 2 | How often do you eat egg in the current pregnancy, till the day you visit this hospital? | 1. Daily 2. __________per week 3. __________ per 2 week 4. __________ per 3 week 5. ___________ per month 6. Not at all |
| 3 | How often do you eat fish during your pregnancy, till the day you visit this hospital? | 1. Daily 2. __________per week 3. __________ per 2 week 4. __________ per 3 week 5. ___________ per month 6. Not at all |
| 4 | How often do you eat legumes (nut, beans, peas,chickpea, soybeans, lentils) during your pregnancy, till the day you visit this hospital? | 1. Daily 2. __________per week 3. __________ per 2 week 4. __________ per 3 week 5. ___________ per month 6. Not at all |
| ,5 | How often do you eat liver and other red meat in the current pregnancy, till the day you visit this hospital? | 1. Daily 2. __________per week 3. __________ per 2 week 4. __________ per 3 week 5. ___________ per month 6. Not at all |
| 6 | How often do you eat citrus fruits in the current pregnancy, till the day you visit this hospital? | 1. Daily 2. __________per week 3. __________ per 2 week 4. __________ per 3 week 5. ___________ per month 6. Not at all |
| 7 | How often do you eat one of the following vegetables (beets, avocados, and potatoes) in the current pregnancy, till the day you visit this hospital? | 1. Daily 2. __________per week 3. __________ per 2 week 4. __________ per 3 week 5. ___________ per month 6. Not at all |
| 8 | How often do you often use iodized salt in your food in the current pregnancy, till the day you visit this hospital? | 1. No 2. Sometimes 3. Always |
| 9 | How often do you use garlic in your food/drink in the current pregnancy, till the day you visit this hospital? | 1. No 2. Sometimes 3. Always |
| 10 | How often do you often use ginger in your food/drink in the current pregnancy, till the day you visit this hospital? | 1. No 2. Sometimes 3. Always |
| 11 | Do you have habits of eating small and more frequent meals, besides of typical meal time (i.e. other than breakfast, lunch, dinner) in the current pregnancy till the day you visit this hospital? | 1. Yes 2. No |
| 12 | Do you have habits of drinking one of the following caffeine-containing substances (coffee, tea, and coca) in the current pregnancy, till the day you visit this hospital? | 1. Yes 2. No |
| 13 | Do you have a habit of eating spiced foods that have intensified flavor by herbs, and spices in the current pregnancy, till the day you visit this hospital? | 1. Yes 2. No |
| 14 | Do you have a habit of saturated fat (solid oils, butter) intake in the current pregnancy, till the day you visit this hospital? | 1. Yes 2. No |
| 15 | How much glass of water you drink per day in the current pregnancy, till the day you visit this hospital? | _________ |

**I have finished thank you very much!!**

**Amharic version questionnaire**

**አባሪ ሁለት: - ለከባድ ማቅለሽለሽ እና ማስታወክ ወሳኝ ነገሮች የመረጃ ማሰባሰቢያ ቅጽ**

የመረጃ ሰባሳቢ ስም _______________ ፊርማ___________

የተሳታፊዎች ቡድን ______________

Case

Control

የመጠይቅ ኮድ _______________

የጤና ተቋም ስም _________________________

**ክፍል አንድ：ማህበራዊና ስነ-ህዝብ ነክ ጥያቄዎች**

| ተራ ቁጥር | ጥያቄ | ምላሽ |
| --- | --- | --- |
| 1 | ዕድሜሽ ስንት ነው? | _______ዓመት |
| 2 | መኖሪያሽ የት ነው? | 1. ከተማ 2. ገጠር |
| 3 | ብሄርሽ ምንድን ነው? | 1. ጋሞ 2. ጎፋ 3. ወላይታ 4. ኮንሶ 5. ኦሞ 6. አምሃራ 7. ሌሎች |
| 4 | ሃይማኖትሽ ምንድን ነው? | 1. ኦርቶዶክስ  2. ሙስሊም  3. ፕሮቴስታንት  4. ሌሎች |
| 5 | የጋብቻ ሁኔታ ? | 1. ያላገባች 2. ያገባች 3. ተለያይተው የሚኖሩ 4. የተፋታች 5. ባሏ የሞተባት |
| 6 | ባልሽ ካንች በተጨማሪ ሌላ ሚስት አለው？ | 1. አዎ 2. አይደለም |
| 7 | ስራሽ ምንድን ነው？ | 1. የቤት እመቤት 2. ተማሪ 3. ነጋዴ 4. የመንግስት ሰራተኛ 5. ገበሬ 6. የቀን ሰራተኛ |
| 8 | የ ትምህርት ደረጃሽ ስንት ነው？ | 1. መደበኛ ትምህርት የለም 2. 1-8 ኛ 3. 9-12 ኛ 4. ኮሌጅ እና ከዚያ በላይ |
| 11 | በትርፍ ጊዜዎት ምን ያክል ጊዜ የአካል ብቃት እንቅስቃሴ ያደርጋሉ? |  |

**ክፍል ሁለት: ከሥነ ተዋልዶ ጋር የተያያዙ ጥያቄዎች**

| ተራ ቁጥር | ጥያቄ | ምላሽ |
| --- | --- | --- |
| 1 | የጽንስ እድሜ በሳምንት ስንት ነዉ？ | ——— ሳምንት |
| 2 | ያሁኑ ስንትተኛ ስንተኛ ዕርግዝናሽ ነው? | ________ |
| 3 | ስንት ጊዜ ወልደሻል? | 1. አልወለድኩም 2. አንድ ጊዜ 3. ከአንድ በላይ |
| 4 | ባሁኑ ወቅት በሄዎት ያሉት ልጆች ስንት ናቸው? | _____ |
| 5 | ባንድ እርግዝና ከአንድ በላይ ጽንሰት አጋጥሞዎት ያዉቃል? በተጨማሪ ካርድ ላይ የተመዘገበ ካለ? | 1. አዎ 2. አይደለም |
| 6 | በቀድሞው እና በአሁኑ እርግዝና መካከል ያለው የጊዜ ቆይታ ስንት ነው? | __________ ወር |
| 7 | ከዚህ በፊት ባዕድ እርግዝና （molar pregnancy) (የበረዶ ግግር/የወይንፍሬ የመሰለ ቅርጽ ያለዉ ጽንስ ) አጋጥሞዎት ያዉቃል ወይም በህክምና ተረጋግጧል? በተጨማሪ ካርድ ላይ የተመዘገበ ካለ？ | 1. አዎ 2. አይደለም |
| 8 | ቀደም ባሉት ጊዜያት በእርግዝና ወቅት የደም ግፊት አጋጥሞዎት ያዉቃል? በተጨማሪ ካርድ ላይ የተመዘገበ ካለ? | 1. አዎ 2. አይደለም |
| 9 | በቀደመው እርግዝና ውስጥ ከባድ ማቅለሽለሽና እና ማስመለስ አጋጥሞዎት ያዉቃል? በተጨማሪ ካርድ ላይ የተመዘገበ ካለ? | 1. አዎ 2. አይደለም |
| 10 | እርግዝናዎ የታቀደ ነው( ከ ባለቤትሽ/ከፍቅር ጓደኛሽ ጋር በጋራ ልጅ ለመዉለድ አቅዳችሁ ተስማምታችሁ ነበር ያረገዝሽዉ? | 1. አዎ 2. አይደለም |
| 11 | እርግዝናዎ ይፈለጋል（ባለቤትሽም አንችም ልጅ የመዉለድ ፍላጎት ነበራችሁ? | 1. አዎ 2. አይደለም |
| 12 | እርግዝናዎ ከትዳር አጋር/ከፍቅረኛዎ እና ከቤተሰብና ድጋፍ አግኝቷል（ ባለቤትሽ/ቤተሰቦችሽ የሞራል/የገንዘብ ድጋፍ እና እንክብካቤ ያደርጉልሽል? | 1. አዎ 2. አይደለም |
| 13 | ካሁን በፊት አርግዘዉ ከነበር በቀደመው እርግዝናዎ ከሚከተሉት ክስተቶች መካከል ቢያንስ አንዱ አጋጥመውዎት ነበር (ፅንስ ማስወረድ ፣ ቀኑሳይደርስ መወለድ ፣ ሞቶ መወለድ ፣ የማህጸንዉስጥ እድገት መገደብ፣የጩቅላ ሕፃናት ሞት ፣ ሙሉ አካል የሌለው ልጅ መዉለድ） | 1. አዎ 2. አይደለም |

**ክፍል ሦስት: ከእናቶች የሕክምና ጉዳዮች ጋር የተያያዙ ጥያቄዎች**

| ተራ ቁጥር | ተራ ቁጥር | | | | ምላሽ | | | |
| --- | --- | --- | --- | --- | --- | --- | --- | --- |
| 1 | በህክምና የተረጋገጠ የስኳር በሽታ ነበረብሽ ? በተጨማሪ ካርድ ላይ የተመዘገበ ካለ | | | | 1. አዎ 2. አይደለም | | | |
| 2 | በህክምና የተረጋግጠ ሃይፐርታይሮይድ በሽታ ነበረብሽ በረብዎት? በተጨማሪ ካርድ ላይ የተመዘገበ ካለ | | | | 1. አዎ 2. አይደለም | | | |
| 3 | የአስም ወይም ሌሎች የመተንፈሻ አካላት ችግር ነበረብሽ? በተጨማሪ ካርድ ላይ የተመዘገበ ካለ? | | | | 1. አዎ 2. አይደለም | | | |
| 4 | በተሽከርካሪ ስትጓዝ ማቅለሽለሽ/ወይም ማስታወክ ይቀስቀስብሽ ነበር? | | | | 1. አዎ 2. አይደለም | | | |
| 5 | | ድብርት | በጭራሽ አይደለም | ብዙ ቀናት | | ከግማሽ ቀናት በላይ | በየቀኑ ማለት ይቻላል |  |
|  | | ስራዎትሽን ለማከናወን ምን ያህል ጊዜ ፍላጎት መቀነስ ይሰማሽ ነበር？ |  |  | |  |  |  |
|  |  | ምን ያህል ጊዜ ተስፋ የመቁረጥ ስሜት ይሰማሽ ነበር? |  |  | |  |  |  |
|  |  | እንቅልፍ ማጣት ወይም ከመጠን በላይ መተኛት ችግር ምን ያህል ጊዜ አጋጥሞሻል？ |  |  | |  |  |  |
|  |  | ምን ያህል ጊዜ የድካም ወይም የኃይል መቀነስ ስሜት ይሰማሽ ነበር? |  |  | |  |  |  |
|  |  | ምን ያህል ጊዜ ደካማ የምግብ ፍላጎት ወይም ከመጠን በላይ የመብላት ችግር አጋጥሞሽ ነበር? |  |  | |  |  |  |
|  |  | ምን ያህል ጊዜ ስለራስሽ መጥፎ ስሜት ይሰማሽ/ ወይም እራስሽን ወይም ቤተሰብሽን እንዳዋረድሽ አድርገሽ ታስቢ ነበር? |  |  | |  |  |  |
|  |  | ምን ያህል ጊዜ የሃሳብ መሰረቅ ወይም ትኩረት ማነስ ችግር ገጥሞሽ ነበር？ |  |  | |  |  |  |
|  |  | ሌሎች ሰዎች ሊያስተውሉት በሚችሉት ሁኔታ በዝግታ የመንቀሳቀስ ወይም መናገር ምን ያህል ጊዜ አጋጥሞሻል? |  |  | |  |  |  |
|  |  | ምን ያህል ጊዜ ብሞት ይሻላል ወይም በሆነ መንገድ እራሴን ብጎዳ ይሻላል ብለሽ አስበሽ ታዉቂያለሽ? |  |  | |  |  |  |

**ክፍል አራት：ከ ሥነ _ልቦና ጋር ተያያዝ ጥያቄዎች**

| ተራ ቁጥር | ጥያቄ | መልስ | | | | |
| --- | --- | --- | --- | --- | --- | --- |
| 1 | የጭንቀት በሽታ | በጭራሽ | አብዛኟው ጊዜ አላጋጠመኝም | አንዳንድ ጊዜ | ብዙ ጊዜ | ሁል ጊዜ |
|  | ባለፈው ወር ባልታሰበ ነገር ሳቢያ ምን ያህል ጊዜ ተበሳጭተዉ ያዉቃሉ? |  |  |  |  |  |
|  | ባለፈው ወር ሕይወትዎ ውስጥ አስፈላጊ ነገሮችን መቆጣጠር ባለመቻልሽ ምን ያህል ጊዜ ተሰምቶሽ ያውቃል? |  |  |  |  |  |
|  | ባለፈው ወር ምን ያክል ጊዜ የመረበሽ እና ጭንቀት ስሜት ይሰማሽ ነበር? |  |  |  |  |  |
|  | ባለፈው ወር የግል ችግሮችን ለመቋቋም ምን ያህል ጊዜ በራስ የመተማመን ስሜት ነበረሽ? |  |  |  |  |  |
|  | ባለፈው ወር ምን ያህል ጊዜ ነገሮች አንች በፈለግሽዉ መንገድ እየተከናወኑ እንደሆነ ይሰማሽ ነበር？ |  |  |  |  |  |
|  | ባለፈው ወር ምን ያክል ጊዜ ነገሮችን ሁሉ መቋቋም የማትችይበት ሁኔታ ላይ እንደሆንሽ ይሰማሽ ነበር? |  |  |  |  |  |
|  | ባለፈው ወር ምን ያህል ጊዜ ብስጭችን/ንዴትን መቆጣጠር ችለሻል？ |  |  |  |  |  |
|  | ባለፈው ወር በስራዎችሽ የስኬት ማማ እንደሆንሽ ምን ያክልጊዚ ይሰማሽ ነበር？ |  |  |  |  |  |
|  | ባለፈው ወር ከቁጥጥርዎ ውጭ በሆኑ ነገሮች ምክንያት ምን ያክል ጊዜ ተቆጥተሻል? |  |  |  |  |  |
|  | ባለፈው ወር ምን ያህል ጊዜ ማሸነፍ እስኪያቅትዎ ድረስ ችግሮች እንደበዙ ተሰምቶሻል? |  |  |  |  |  |
| 2 | ከአሁን በፊት መጥፎ የእርግዝና አጋጣሚዎች(ፅንስ ማስወረድ ፣ ቀኑሳይደርስ መወለድ ፣ ሞቶ መወለድ ፣ የሕፃናት ሞት፣ ሙሉ አካል የሌለው ልጅ መዉለድ） አጋጥሞዎት የሚያቅ ከሆነ ያጋጠመዎት ክስተት በአሁኑ እርግዝና በድጋሜ ይከሰታል ብለዉ ይፈራሉ? | 1. አዎ 2. አይደለም |  |  |  |  |

**ክፍል አምስት-ከአመጋገብ ጋር ተያያዥ የሆኑ ጉዳዮች**

| ተራ ቁጥር | ጥያቄ | ምላሽ |
| --- | --- | --- |
| 1 | በአሁኑ እርግዝናሽ ወቅት ወደዚህ ጤና ተቋም እስከመጣሽበት ቀን ድረስ ወተት/የወተት ተዋጽኦዎችን ምን ያህል ጊዜ ትመገቢ ነበር? | 1. በየቀኑ 2. —— ጊዜ በሳምንት 3. —— ጊዜ በ 2 ሳምንት 4. —— ጊዜ በ 3 ሳምንት 5. —— ጊዜ በ ወር 6. በጭራሽ አልተመገብኩም |
| 2 | በአሁኑ እርግዝናሽ ወደዚህ ጤና ተቋም እስከመጣሽበት ቀን ድረስ እንቁላል ምን ያህል ጊዜ ትመገቢ ነበር? | 1. በየቀኑ 2. —— ጊዜ በሳምንት 3. —— ጊዜ በ 2 ሳምንት 4. —— ጊዜ በ 3 ሳምንት 5. —— ጊዜ በ ወር 6. በጭራሽ አልተመገብኩም |
| 3 | በአሁኑ እርግዝናሽ ወደዚህ ጤና ተቋም እስከመጣሽበት ቀን ድረስ ዓሳ ምን ያህል ጊዜ ትመገቢ ነበር? | 1. በየቀኑ 2. —— ጊዜ በሳምንት 3. —— ጊዜ በ 2 ሳምንት 4. —— ጊዜ በ 3 ሳምንት 5. —— ጊዜ በ ወር 6. በጭራሽ አልተመገብኩም |
| 4 | በአሁኑ እርግዝናሽ ወደዚህ ጤና ተቋም እስከመጣሽበት ቀን ድረስ ጥራጥሬዎችን(ለዉዝ፣ባቄላ፣አተር፣ሽምብራ፣አኩሪያአተ፣ምስር） ምን ያህል ጊዜ ትመገቢ ነበር?? | 1. በየቀኑ 2. —— ጊዜ በሳምንት 3. —— ጊዜ በ 2 ሳምንት 4. —— ጊዜ በ 3 ሳምንት 5. —— ጊዜ በ ወር 6. በጭራሽ አልተመገብኩም |
| 5 | በአሁኑ እርግዝናሽ ወደዚህ ጤና ተቋም እስከመጣሽበት ቀን ድረስ ጉበት ወይም ሌሎች ቀይ ስጋ ምን ያህል ጊዜ ትመገቢ ነበር？ | 1. በየቀኑ 2. —— ጊዜ በሳምንት 3. —— ጊዜ በ 2 ሳምንት 4. —— ጊዜ በ 3 ሳምንት 5. —— ጊዜ በ ወር 6. በጭራሽ አልተመገብኩም |
| 6 | በአሁኑ እርግዝናሽ ወደዚህ ጤና ተቋም እስከመጣሽበት ቀን ድረስ ኮምጣጤ ፍራፍሬዎችን（ሎሚ፣ብርቱካን፣ አፕል፣ ሌላም） ምን ያህል ጊዜ ትመገቢ ነበር? | 1. በየቀኑ 2. —— ጊዜ በሳምንት 3. —— ጊዜ በ 2 ሳምንት 4. —— ጊዜ በ 3 ሳምንት 5. —— ጊዜ በ ወር 6. በጭራሽ አልተመገብኩም |
| 7 | በአሁኑ እርግዝናሽ ወደዚህ ጤና ተቋም እስከመጣሽበት ቀን ድረስ ከሚከተሉት አትክልቶች ውስጥ ቢያንስ አንዱን (ቀይ ስር ፣ አቮካዶ እና ድንች) ምን ያህል ጊዜ ትመገቢ ነበር？ | 1. በየቀኑ 2. —— ጊዜ በሳምንት 3. —— ጊዜ በ 2 ሳምንት 4. —— ጊዜ በ 3 ሳምንት 5. —— ጊዜ በ ወር 6. በጭራሽ አልተመገብኩም |
| 8 | በአሁኑ እርግዝናሽ ወደዚህ ጤና ተቋም እስከመጣሽበት ቀን ድረስ አዮዲን ያለው ጨው（ የታሸገ ጨዉ）ለምግብ መስሪያነትምን ያህል ጊዜ ትጠቀሚ ነበር? | 1. በጭራሽ አልተጠቀምኩም 2. አልፎ አልፎ 3. ሁልጊዜ |
| 9 | በአሁኑ እርግዝናሽ ወደዚህ ጤና ተቋም እስከመጣሽበት ቀን ድረስ ነጭ ሽንኩርት በምግብ / በመጠጥዎ ውስጥ ምን ያህል ጊዜ ትጠቀሚ ነበር? | 1. በጭራሽ አልተጠቀምኩም 2. አልፎ አልፎ 3. ሁልጊዜ |
| 10 | በአሁኑ እርግዝናሽ ወደዚህ ጤና ተቋም እስከመጣሽበት ቀን ድረስ ዝንጅብል በምግብ / በመጠጥዎ ውስጥ ምን ያህል ጊዜ ትጠቀሚ ነበር? | 1. በጭራሽ አልተጠቀምኩም 2. አልፎ አልፎ 3. ሁልጊዜ |
| 11 | በአሁኑ እርግዝናሽ ወደዚህ ጤና ተቋም እስከመጣሽበት ቀን ድረስ መክሰስ（በ ቁርስ እና በ ምሳ፣በምሳ እና በ እራት መካከል） የመመገብ ልምድ ነበረሽ? | 1. አዎ 2. አይደለም |
| 12 | በአሁኑ እርግዝናሽ ወደዚህ ጤና ተቋም እስከመጣሽበት ቀን ድረስ ከከሚከተሉት አንዱን መጠጥ ቢያንስ በቀን አንድ ጊዜ (ቡና ፣ ሻይ እና ኮካ) የመጠጣት ልምድ ነበረሽ? | 1. አዎ 2. አይደለም |
| 13 | በአሁኑ እርግዝናሽ ወደዚህ ጤና ተቋም እስከመጣሽበት ቀን ድረስ የምግብ ሽታን ለማጎልበት/ቅመማቅመም እና ቅጠላቅጡሎችን በምግብ ላይ ጨምረሽ ትመገቢ ነበር？ | 1. አዎ 2. አይደለም |
| 14 | በአሁኑ እርግዝናሽ ወደዚህ ጤና ተቋም እስከመጣሽበት ቀን ድረስ የሚረጋ/ቀላጭ ዘይትን ወይም ቅቤን ለምግብ ማጣፈጫነት ትጠቀሚ ነበር? | 1. አዎ 2. አይደለም |
| 15 | በአሁኑ እርግዝናሽ ወደዚህ ጤና ተቋም እስከመጣሽበት ቀን ድረስ በየቀኑ ምን ያህል ብርጭቆ ውሃ ትጠጭ ነበር? | __________ |
