# Supplementary material for: Determinants of hyperemesis gravidarum among pregnant women attending health care service in public hospitals of Southern Ethiopia
Source: PLoS One. 2022 Apr 26;17(4):e0266054. doi: 10.1371/journal.pone.0266054 (PMC9042275; doi:10.1371/journal.pone.0266054)
Supplement: Attachment — Submitted filename: Comments-1.docx [file pone.0266054.s003.docx]

Comments

Re-write the title as

Determinants of hyperemesis gravidarum among pregnant women attending health care service in public hospitals of Southern Ethiopia: a case control study, then you should clearly present the study areas either in sentence or schematic under the methodology section.

Line 21-22: starting before the end of the 22nd week of gestation, please write when hyperemesis starting and when becomes peak por worsening.

Line 22-23: The identification of the determinants of hyperemesis gravidarum is of particular importance, Have full of typo editing problems

Line 24-25: It lacks coherence, albeit it should be modified

In Ethiopia there is low information on determinants of hyperemesis gravidarum, I don’t think so, how you are sure, the information is low regarding HG, please come up with any evidence.

Why you want to carried out using unmatched case control, studies showed that most of the risk factors are unknown, however some modifiable risk factors are already investigated, as such it is better to make matched either in gestational age, age of the patient, number of parity or any others, otherwise it doesn’t sounds good.

What does mean structured questionary??

Hope, you used 1 to 2 ration, what is the principle and rule of thumb for the ratio selection and why you choose 1 to 2.

Please avoid merging different medical disorders, because it leads to confounding. For eg. having history asthma/ other respiratory tract infections. Do you think the effect of all respiratory tract infections are similar, if not compute separately?

What things you recommend for thus factors; urban residence, having polygamous husband

You stated as: Despite having many studies done elsewhere, in Ethiopia little is known about determinants of hyperemesis gravidarum, what things odd in Ethiopia, unless you are not going to study any genetics analysis or race based analysis.

I think you used different hospitals such as General and Primary, how do you see the psychological management of HG in general and primary hospitals may be consistent, either professional skills, availability and environmental factors,

Please shorten the study area and period as a manuscript, don’t copy paste the original thesis as it is.

On source and study population, it needs language refinement.

Please look at seriously (All pregnant women in the antenatal period attending health care), rephrase and modify it

Eg. All pregnant women who attended Antenatal care in public hospitals of Gamo Gofa, and south Omo zone

Modify the study population for cases and controls as I mentioned above.

What is your case and control definition???? Make sure that it is clear, how do you do if they are falsely reported.

Please see carefully your exclusion criteria, what does it mean severely ill. In addition, rewrite the sentence

Why you excluded ever treated for HEG in the current pregnancy?

Please write the heading as sample size determination and sampling techniques, then shorten and write the key information’s only.

Avoid variables of the study, it is optional, but not this much important for high quality journals

How do you measure and which techniques you used to assess psychological factors like fear of reoccurrence of BOH

If you are going to study dietry factors in the first time in Ethiopia, what type of tools you used?

I’m in doubt, your study has had multi collinearity, have you assed , if yes what technique and how you ruled out, because under your regression table the AOR is higher than the COR, particularly for residence and having polygamous husband

Please cite, the reason for your discussion, if it is biological plausible and already used from other literatures

Overall the finding id nothing adds any new literature to the world, at this time in low income countries better to study management modalities, perinatal outcomes and complications of HG.

You concluded, what any books recommends, everything is clearly known. Sorry I can’t able to catch-up new findings.
