# Supplementary material for: Determinants of hyperemesis gravidarum among pregnant women attending health care service in public hospitals of Southern Ethiopia
Source: PLoS One. 2022 Apr 26;17(4):e0266054. doi: 10.1371/journal.pone.0266054 (PMC9042275; doi:10.1371/journal.pone.0266054)
Supplement: Attachment — Submitted filename: Response to reviewer(s).docx [file pone.0266054.s004.docx]

**Author’s Point-by-Point Response to the Reviewer's and Editors Reports**

**Title**: **Determinants of hyperemesis gravidarum among pregnant women attending health care service in public hospitals, Southern Ethiopia.**

**Corresponding author**: Kassaw Beyene / Kassaw.kb3@gmail.com

PONE-D-21-31620

Journal: PLOS ONE
**Point by point response to Reviewers and Editors**

First of all, the authors would like to thank PLOS ONE Journal editors and the respective reviewers for reviewing this manuscript and providing the necessary comments to be corrected. As per the comments given, we have made modifications and presented point by point to each comment. The authors tried to answer all the issues raised by editorial team and reviewers. Please note that we gave the response in blue font color.

**Reviewer 1: comments and the response given from the authors.**

Comment 1: Re write the tittle as Determinants of hyperemesis gravidarum among pregnant women attending health care service in public hospitals of Southern Ethiopia: a case control study

Response 1: Thank you very much for this suggestion. As per your suggestion it was already corrected, we kindly invited you to check either from the track change or from clean manuscript

Comment 2: Line 21-22: starting before the end of the 22nd week of gestation, please write when hyperemesis starting and when becomes peak poor worsening.

Response 2: Thank you for your interesting comments, it was corrected

Comment 3: Line 22-23: The identification of the determinants of hyperemesis gravidarum have particular importance, Have full of type editing problems

Response 3: All the comments given in each line by line as well as the grammar part and way of writing style was modified and corrected accordingly. The authors kindly request the reviewer to appreciate the modification from the track change and corrected manuscript.

Comment 5: In Ethiopia there is low information on determinants of hyperemesis gravidarum, I don’t think so, how you are sure, the information is low regarding HG, please come up with any evidence.

Response 5: Actually there are some studies conducted in Ethiopia (in Addis Ababa, Arba Minch and Bale Robe ) both studies conducted in Addis Ababa and Arba Minch were descriptive and almost all recommends to determine the determinant factors of HEG, we our study may address their suggestion and fill gaps in which the previous studies lost.

Comment 6: Why you want to carried out using unmatched case control, studies showed that most of the risk factors are unknown, however some modifiable risk factors are already investigated, as such it is better to make matched either in gestational age, age of the patient, number of parity or any others, otherwise it doesn’t sounds good.

Response 6: thank you very much for this constructive suggestion, from the beginning the authors consider to conduct match case control but it is difficult to get matched controls for case mainly by their gestational age due to the fact that pregnant women come to hospital after five month if the they are healthy, and it makes difficult to access control.

Comment 7: What does mean structured questionary?

Response 7: A structured questionnaire in which the interviewer asks a particular set of predefined questions that are planned and created in advance, which means that all the respondents asked the same questions and in the same order.

Comment 8: Hope, you used 1 to 2 ration, what is the principle and rule of thumb for the ratio selection and why you choose 1 to 2.

Response 8: The rule of thumb for case and controls is maximum 1:4 and minimum 1:1, in our scenario since the case( hyperemesis gravidarum) is rare disease to maximize our sample size and increase the power of the study and considering resource(time and money) constraint, that is why we used 1:2 ratio.

Comment 9: Please avoid merging different medical disorders, because it leads to confounding. For eg. having history asthma/ other respiratory tract infections. Do you think the effect of all respiratory tract infections are similar, if not compute separately?

Response 9: Actually you are right, but the aim of assenting respiratory tract disorders is to see the mechanical effects cough on GIT secondary to respiratory diseases, almost all the respiratory problems that the study assessed have cough as a result to see its effect on GIT.

Comment 10: What things you recommend for thus factors; urban residence, having polygamous husband

Response 11: as per your suggestion all associated factors are not recommended particularly for implementation like residence but factor like having polygamous husband is recommended to her family members/ relatives to give care and support for pregnant women who have polygamous husband even if this practice is deep rooted in the culture in the study area

Comment 11: You stated as: Despite having many studies done elsewhere, in Ethiopia little is known about determinants of hyperemesis gravidarum, what things odd in Ethiopia, unless you are not going to study any genetics analysis or race based analysis.

Response 12: Thank you very much for this constructive suggestion, even though the study was conducted vigorously at the Globe those study finding are not applicable for us because we have different cultures, life style, dietary practice during pregnancy, physical activity during pregnancy and socio demographic difference, So those factors make Ethiopia unique beside genetic/ race difference.

Comment 12: I think you used different hospitals such as General and Primary, how do you see the psychological management of HG in general and primary hospitals may be consistent, either professional skills, availability and environmental factors,

Response 13: I appreciate your concern, institutional level, professional skill, availability of material and expert and environmental factors may affect the prognosis of HEG rather than the exposure status, but since our study is case-control we intend to identify the determinants of HEG, simply we assess the exposure status of the pregnant women.

Comment 13: Please shorten the study area and period as a manuscript, don’t copy paste the original thesis as it is.

Response 13: Thank you very much for this constructive suggestion, based on your suggestion the study area and period were shorten. You can kindly check the modification in the clean manuscript and manuscript with track change.

Comment 14: On source and study population, it needs language refinement.

Response 14: Thank you for your suggestion, we have corrected as per your suggestion. You can kindly check the modification either from the clean manuscript or manuscript with track change.

Comment 15: Please look at seriously (All pregnant women in the antenatal period attending health care), rephrase and modify it

Response 15: Thank you for your interesting comments, as per your comments the study populations were rephrased and modified. You can kindly check the modification in the clean manuscript and manuscript with track change.

Comment 16: Modify the study population for cases and controls as I mentioned above.

Response 16: Thank you for your interesting comments, as per your comments the study populations were rephrased and modified. You can kindly check the modification in the clean manuscript and manuscript with track change.

Comment 17: What is your case and control definition???? Make sure that it is clear, how do you do if they are falsely reported.

Response 17: As stated in the operational definition ‘**Cases**: women in antenatal period clinically diagnosed by the physician as being hyperemesis gravidarum.

**Controls**: women in the antenatal period that had not been diagnosed with hyperemesis gravidarum’. From the begging in order not to have false report beside the diagnosis of the case by health care providers, we have provided intensive training for data collectors and supervisors.

Comment 18: Please see carefully your exclusion criteria, what does it mean severely ill. In addition, rewrite the sentence

Response 18: Thank you for this constructive suggestion, based on this study context severely ill means patient who are diseased and unable respond for the interview due the seriousness of the illness during the study period, so those patients are excluded from the study if they are not capable to give response.

Comment 19: Why you excluded ever treated for HEG in the current pregnancy?

Response 19: thank you for this concern, those pregnant women who are treated for HEG were excluded from the control because those women may become cases since the recurrence rate of HEG high and except the gestational age during the interview period, the women will give the same response as if she was interviewed during her illness, and she already develop the case before the study started.

Comment 20: Please write the heading as sample size determination and sampling techniques, then shorten and write the key information’s only.

Response 20: thank you for your comments, it was corrected and you can kindly check the modification in the clean manuscript and manuscript with track change.

Comment 21: Avoid variables of the study, it is optional, but not this much important for high quality journals

Response 21: variables were avoided and corrected

Comment 22: How do you measure and which techniques you used to assess psychological factors like fear of reoccurrence of BOH

Response 22: Thanks, just a single question was used to assess the fear of reoccurrence of bad obstetrics history , if she had previous history.

Comment 23: If you are going to study dietary factors in the first time in Ethiopia, what type of tools you used?

Response 23: Thank you very much for your depth review, To assess the dietary factors dietary history was asked by using **food frequency sheet** which is used by other studies other than HEG.

Comment 24: I’m in doubt, your study has had multi collinearity, have you assed , if yes what technique and how you ruled out, because under your regression table the AOR is higher than the COR, particularly for residence and having polygamous husband

Response 24: Thank you for the concern, Yes multi-collinearity was checked by using VIF: but it advisable to do multi-collinearity test for continuous variable

Comment 25: Please cite, the reason for your discussion, if it is biological plausible and already used from other literatures

Response 25: Thank you, we have seen back the discussion section all discussed points were cited.

Comment 26: Overall the finding id nothing adds any new literature to the world, at this time in low income countries better to study management modalities, perinatal outcomes and complications of HG.

Response 26: we hope, this study adds some inputs that strength the existing studies finding regarding the determinants of HEG particularly in Ethiopia, different studies were done in different corner of the world but those findings are not sharply meet with our contexts, since we have different dietary habit, medical checkup, psychological support, physical activity and life style e t c.

Comment 27: You concluded, what any books recommends, everything is clearly known. Sorry I can’t able to catch-up new findings.

Response 27: Our conclusion is made based on the study finding and revealed that which factors determine the occurrence of HEG, and this finding support and contribute a piece of ideas for the scientific community mainly being studied in Ethiopia context.
