# Supplementary material for: Determinants of hyperemesis gravidarum among pregnant women attending health care service in public hospitals of Southern Ethiopia
Source: PLoS One. 2022 Apr 26;17(4):e0266054. doi: 10.1371/journal.pone.0266054 (PMC9042275; doi:10.1371/journal.pone.0266054)
Supplement: Attachment — Submitted filename: Response to Reviewers.docx [file pone.0266054.s005.docx]

**Author’s Point-by-Point Response to the Reviewer's and Editors Reports**

**Title**: **Determinants of hyperemesis gravidarum among pregnant women attending health care service in public hospitals, Southern Ethiopia.**

**Corresponding author**: Kassaw Beyene / Kassaw.kb3@gmail.com

PONE-D-21-31620

Journal: PLOS ONE
**Point by point response to Reviewers and Editors**

First of all, the authors would like to thank PLOS ONE Journal editors and the respective reviewers for reviewing this manuscript and providing the necessary comments to be corrected. As per the comments given, we have made modifications and presented point by point to each comment. The authors tried to answer all the issues raised by editorial team and reviewers. Please note that we gave the response in blue font color.

**Reviewer’s comment and the response given from the authors.**

Abstract
1. The background section of the abstract requires reconsideration. For instance, how studying the determinants of HEG could enhance early detection of HEG? In fact, it may be beneficial for early intervention to prevent occurrence of HEG, and further health and other damages once it happens.

Response 1: thank you for your concern, the intention of the paper is if the determinants of HEG is identified early it helps us to intervene early before the condition worsen and may result in psychological, medical and economical burden on the women, her family and health care system.

2. What is the implication of having polygamous husband and HEG? How could you justify this relationship?

Response 2: thank you for your concern, in the study area having polygamous husband is deeply rooted in the culture, women with polygamous husband are highly exposed for loss of psychological, dietary (economical) and physical support, due to this reason women having polygamous husband were exposed to have HEG in their pregnancy, it is recommended to her family members/ relatives to give care and support for pregnant women who had polygamous husband.
3. Line 24… delete coma: “psychosocial and, economic impact.”

Response 3: Thank you for your suggestion, we have corrected as per your suggestion. You can check the correction either from the clean manuscript or manuscript with track change
4. Line 28…add spacing: “to collect the data from 360 study participants”

Response 4: Thank you for your suggestion, based on your suggestion, it was corrected

5. Line 29…check spelling: “Kobcollect 1.3”. Even in this document I see spelling inconsistency for the same software stated here in the abstract and in the method sections… “Kobkollect”. The correct may be “KoBoCollect…”

Response 5: Thank you for your critical review, it was corrected as per your suggestion. You can kindly check the modification either from the clean manuscript or manuscript with track change
6. Line 35…unnecessary capitalization: “Saturated fat intake”

Response 6: Thank you for your suggestion, we have corrected as per your suggestion. You can kindly check the correction either from the clean manuscript or manuscript with track change

7. The authors should rephrase the recommendation. The role that the government authorities have to play and even the word itself is vaguely stated. Do they really have to involve or expected to involve in health education and counselling?

Response 7: Thank you for your suggestion, we have corrected as per your suggestion, we intend to recommend the Government authority particularly FDA authority to plan a strategy to conduct health education about the effect of using saturated fat via different communication channels/ mass media.
Introduction
8. The introduction is not well synthesized and lacks coherence. For example, the first paragraph is long and taken from a single reference. Another example, 4th and 5th paragraphs are about risk factors of HEG while 6th and 7th paragraphs are about consequences of HEG. In the 8th paragraphs the authors come again to discuss about risk factors of HEG.

Response 8: Thank you for your suggestion, we have corrected as per your suggestion as much as possible. You can kindly check the modification either from the clean manuscript or manuscript with track change
9. The authors didn’t show the magnitude of HEG in Ethiopia in general and in study area in particular. Despite knowing the consequential outcomes of HEG, understand the magnitude of HEG

Response 9: Thank you for your suggestion, we have corrected as per your suggestion. You can kindly check the modification either from the clean manuscript or manuscript with track change is very helpful for readers to have clear image in study area context.
10. Line 64 through 72 are about risk factors of HEG. But the evidences are not synthesized. The authors presented the contents of each sources cited to make up an independent sentence.

Response 10: Thank you for your suggestion, we have corrected as per your suggestion. You can kindly check the modification either from the clean manuscript or manuscript with track change.
11. The arguments given in the last paragraph are not convincing. Even the factors are presented very grossly…sociodemographic factors, medical factors, obstetric factors, etc. Please try to address them in detail and in narrower scope. The sample size issue is also not clear. Was that not scientifically sound? How that happen? The authors also raise the argument that study areas were not included in the previous study…what is special for the study area? Anything that interests you to study in the stated sites/area?

Response 11: Thank you for your suggestion, we have corrected as per your suggestion. Things that interest us to study in this area is high prevalence of the problem which accounts 8.2% in Arba Minch. And this study which was conducted in Arba Minch previously also recommended to study on the determinant factors of HEG. You can kindly check the modification either from the clean manuscript or manuscript with track change
12. Line 88… vague description: “A similar study also concludes that demographic and obstetric…” The authors say similar study but with different citation, in this case, what is the linking word similar is referring to? In terms of what? Was that in terms of study design or …?

Response 12: Thank you for your suggestion, it was corrected as per your suggestion.
13. Line 90…grammatical concern: “On the contrary, other studies on the contrary conclude that demographic…”

Response 13: Thank you for your suggestion, it was corrected as per your suggestion.
14. The issue of early detection must be reconsidered here also.

Response 14: Thank you, if someone can early identify the risk factors that leads to HEG, it is possible to intervene to alleviate that risk before resulting in HEG and its effect on the women and her family.
**Methods**
15. Too many subheadings, the authors can use relevant subheading to organize the contents under fewer subheadings.

Response 15: Thank you for your constructive review, as much as possible we have corrected as per your suggestion. You can check the correction either from the clean manuscript or manuscript with track change
16. The control definition and exclusion criteria. Did the selection of the controls and cases are done regardless of the gestational age or trimester?

Response 16: Thank you for your concern, yes, the selection of controls and cases are regardless of the gestational age, that is why the study design is unmatched case control. since HEG can be a problem throughout pregnancy, limiting the study participants by gestational age is not acceptable ethically. Those pregnant women who are treated for HEG were excluded from the control because those women may become cases since the recurrence rate of HEG high and except the gestational age during the interview period, the women will give the same response as if she was interviewed during her illness, and she already develop the case before the study started.
17. The author should give citation for the article used to calculate the sample size and also include a description about where the referred study was done. Factors for NVP Vs HEG: were the factors you considered in sample size calculation are for NVP or HEG? If you used factors for NVP, how much they are relevant to the outcome of interest?

Response 17: Thank you for your suggestion, we have corrected as per your suggestion. It was taken from the previous study done in Bale zone, Ethiopia (by taking the factor being in the 2^nd^ trimester when severe NVP or HEG starts). women who have severe nausea and vomiting during her pregnancy is considered as HEG and other studies are taken, so we used interchangeably, but if nausea and vomiting is mild, we can’t use to calculate the sample size for our outcome of interest.
18. Line 104…coma misplaced. It is corrected as “Arba Minch Town, which is the Capital of the Gamo Zone, is 505…”. Similar correction is needed in line 107: “Sawla Town, which is the Capital of Gofa Zone, is 464”. Check line 110 for the same problem.

Response 18: Thanks, we have corrected as per your suggestion. You can kindly check the modification either from the clean manuscript or manuscript with track change
19. Sampling Techniques: Regarding case and control selection, the authors already stated that they used a systematic sampling after proportional allocation. But the K used at each health facility was unique, how this happened is not clear? Proportional allocation Vs Different K?

Response 19: Thank you for your suggestion, to get the required number of cases and controls, proportional allocation was done to each hospital based on the number of women admitted for HEG and the number of women attending ANC visit in the previous time. By default, for Cases the k value was 2 for all hospitals (2 point something for some hospitals and sharply 2 for some hospitals) so every other woman was selected, but for control selection, k value for each hospital was different, because if we take the same k value for all hospitals, we can’t obtain the required number of controls with in the time frame.

20. The author should provide very clear detail about each variable measurement especially those variables having psychometric properties such as stress, depression, etc; variable requiring clinical skill and dietary intake related assessments. The data collection methods used are also required to be clear. Nothing was stated about the data collection methods used in the method section except the crude insight given in the abstract section.

Response 20: Thank you for your suggestion, we have corrected as per your suggestion. All those concerns are addressed under Data collection tool and quality control section. You can kindly check the modification either from the clean manuscript or manuscript with track change
21. The authors should give attention for the Cronbach’s alpha value given in the data quality management section. What is the implication of a Cronbach’s alpha value above 0.90? The reliability given for perceived stress is 0.949.

Response 21: Thank you for your critical review, for our study a Cronbach’s alpha value above 0.90 implies that the internal consistency(reliability) of the tool is strong and highly reliable. But statically, if Cronbach alpha > 0.90 implies that there is redundance of tool and short test length.
22. The author shall provide detail information about data analysis. For example, candidate selection variable strategies for multivariable logistic regression were not explained. The issue of multicollinearity checks before fitting multivariable logistic regression model need to be addressed.

Response 22: Thank you for your suggestion, candidate variable selection criteria for multivariable logistic regression were variables with p value less than or equal to 0.25, biological plausibility and consistency in the previous study, but in our study both strategies, biological plausibility and consistency in the previous study are not applied that is why both strategies are not stated under the method section. And also, multicollinearity test was done before model fitness was assessed and VIF was less than 10.
23. There are editorial problems…unnecessary bolding, inconsistent font size, grammatical issues, check how hyphen used, etc.

Response 23: Thank you for your critical review, as much as possible we have corrected as per your suggestion. You can kindly check the modification either from the clean manuscript or manuscript with track change
Results
24. The authors didn’t show how they come up with these determinants. They should clearly indicate the candidate variables for the multivariable logistic model. Then, they can go for result interpretations as they did now. Overall, how the multivariable logistic model was fitted is overlooked both in the method and in the results sections.

Response 24: Thank you for your suggestion, it was corrected as per your suggestion. You can kindly check the modification either from the clean manuscript or manuscript with track change
25. In Table 4, the authors presented p-values, for what these p-values stands for is my question? Is that for COR or AOR? What is the relevance of having p-value in the presence of confidence intervals for odds ratios? The table design is not even attractive for reader.

Response 25: Thank you for your suggestion, we have corrected as per your suggestion. You can kindly check the modification either from the clean manuscript or manuscript with track change
26. Line 222…error: “A total of 360 study participants (120 cases and 2240 controls)”

Response 26: Thank you for your suggestion, the editorial error is corrected. You can appreciate the modification either from the clean manuscript or manuscript with track change
27. Line 224…spacing: 89(74.2 %)

Response 27: Thank you for your suggestion, we have corrected as per your suggestion. You can kindly check the modification either from the clean manuscript or manuscript with track change
 28. There are many editorial problems… inconsistent font size, grammatical issues, etc.

Response 28: Thank you for your suggestion, as much as possible all your concern were tried to be addressed. You can kindly check the modification either from the clean manuscript or manuscript with track change
Discussion
29. The discussion requires a major revision. It is better if you begin with brief summary of the main findings in the first paragraph. The results are brought here in the discussion without significant paraphrasing. Besides, the possible justifications or the implications of results given for the observed association are not convincing or non-relevant in some cases. Even some of the comparisons are vague to understand. For example, the comparison given for the positive association between urban residence and HEG are studies which conclude about the association between HEG and demographic factors…which demographic factors? Was that about residence? At the same time the justifications given are also not relevant. Make things very clear as much as possible.

Response 29: Thank you for your suggestion, we have corrected as per your suggestion. You can kindly check the modification either from the clean manuscript or manuscript with track change
Conclusion
30. The authors should revise the recommendation.

Response 30: Thank you for your suggestion, as per your suggestion we tried to modify. You can kindly check the modification either from the clean manuscript or manuscript with track change
